# Siderophore–Antibiotic Conjugate Design: New Drugs for Bad Bugs?

**DOI:** 10.3390/molecules24183314

**Published:** 2019-09-11

**Authors:** Kokob H. Negash, James K.S. Norris, James T. Hodgkinson

**Affiliations:** Leicester Institute of Structural and Chemical Biology, School of Chemistry, University of Leicester, George Porter Building, University Road, Leicester LE1 7RH, UK

**Keywords:** siderophore, antibiotic, siderophore–antibiotic, trojan horse, antibiotic resistance

## Abstract

Antibiotic resistance is a global health concern and a current threat to modern medicine and society. New strategies for antibiotic drug design and delivery offer a glimmer of hope in a currently limited pipeline of new antibiotics. One strategy involves conjugating iron-chelating microbial siderophores to an antibiotic or antimicrobial agent to enhance uptake and antibacterial potency. Cefiderocol (S-649266) is a promising cephalosporin–catechol conjugate currently in phase III clinical trials that utilizes iron-mediated active transport and demonstrates enhanced potency against multi-drug resistant (MDR) Gram-negative pathogens. Such molecules demonstrate that siderophore–antibiotic conjugates could be important future medicines to add to our antibiotic arsenal. This review is written in the context of the chemical design of siderophore–antibiotic conjugates focusing on the differing siderophore, linker, and antibiotic components that make up conjugates. We selected chemically distinct siderophore–antibiotic conjugates as exemplary conjugates, rather than multiple analogues, to highlight findings to date. The review should offer a general guide to the uninitiated in the molecular design of siderophore–antibiotic conjugates.

## 1. Introduction

Antimicrobial resistance, including antibiotic resistance, is a major threat to global human health and society at large [1]. The increased incidence of infections with multi-drug resistant (MDR) pathogens is associated with more complex and elaborate drug regimens, extended hospitalization periods, and ultimately increased patient mortality [2]. Although new antibiotics such as teixobactin, with a novel mode of action, offer approaches toward overcoming the antibiotic crisis, the clinical efficacy of such new therapies in patients is yet to be determined. There are also not enough new antibiotics being trialed and ultimately marketed [3]. This existing lack of new antibiotics, combined with bacterial evolution to resist current antibiotics, has led to serious concerns of an eminent return to the ‘pre-antibiotic era′ [1].

Bacterial resistance to antibiotics can occur through a number of mechanisms. Such mechanisms include the overexpression of the antibiotic target enzyme/protein, mutations in the antibiotic target protein/enzyme, overexpression of antibiotic-modifying enzymes such as β-lactamases, and the utilization of multiple efflux pumps to actively export the antibiotic out of the bacterial cell, reducing the intracellular accumulation of the antibiotic [4]. The Gram-negative cell wall also consists of an additional outer membrane posing a significant permeability barrier to the diffusion and efficacy of many antibiotics [5].

One approach to overcome the bacteria cell wall permeability barrier and reduced intracellular accumulation is to covalently link an antibiotic to a microbial siderophore or siderophore mimic [5]. Siderophores are small organic chelators (molecular weight between 150–2000 Da) with high affinity for iron [6]. Iron is an essential requirement of every living organism as a cofactor for a large number of proteins involved in many fundamental and essential cellular processes, including electron transfer, cell respiration, and superoxide metabolism. The solubility of iron at physiological pH in the aerobic environment is very low, as the presence of oxygen oxidizes Fe(II) into insoluble Fe(III) ferric oxyhydroxides [6]. In addition, the concentration of iron is further lowered in the infection microenvironment, as there is a constant battle for the infected host cell to suppress the concentration of iron for bacterial cell import [7].

**Figure 1:**
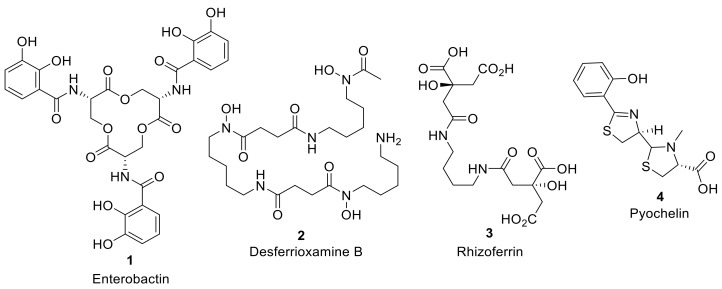
Selected examples of iron-chelating microbial siderophores: Enterobactin (**1**) is a catecholate siderophore produced by Gram-negative bacterial species such as *Escherichia coli* and *Salmonella typhimurium* and one of the highest affinity chelators of Fe (III); Desferrioxamine B (**2**) is a hydroxmate siderophore produced by the Gram-positive bacteria *Streptomyces pilosus* used in iron overdose treatment; Rhizoferrin (**3**) is a carboxylate siderophore isolated from the fungus *Rhizopus microspores*; Pyochelin (**4**) is a mixed siderophore containing both phenolate and carboxylic acid iron chelating groups, produced by the Gram-negative bacterial pathogen *Pseudomonas aeruginosa*.

Siderophores are secreted by bacteria to the extracellular environment to solubilize and import iron, and are especially important in competitive multimicrobial environmental niches and iron-restricted host organisms. More than 500 different siderophores have now been identified with high structural diversity [6]. However, despite this structural diversity, the coordinating functional groups of the siderophore to chelate iron are generally consistent, including: hydroxamates, catechols, carboxylates, phenolate moieties, or indeed mixed combinations of these functional groups, attached to linear or cyclic scaffolds to form, often, a hexadentate chelate structure (Figure 1) [6]. Once iron is chelated in the extracellular environment, the iron–siderophore complex (ferrisiderophores) are recognized by high-affinity receptor proteins in the bacteria cell wall that utilize active transport to internalize the iron [8]. In Gram-negative bacteria, ferrisiderophores cross the outer membrane periplasm, and the inner membrane to reach the cytoplasm, where Fe(III) in the ferrisiderophore is reduced to Fe(II) and released; alternatively, free iron can be released from the ferrisiderophore in the periplasm for internalization to the cytoplasm [8].

A considerable number of studies have shown that siderophore iron uptake pathways can be used to transport siderophore–antibiotic conjugates into the bacterial cell by active transport. This method is often called the ‘Trojan horse′ approach [9]. Astounding results have been achieved, including reduced minimum inhibitory concentration (MIC) values up to a 1000-fold reduction [10], modified spectrum of activity from Gram-positive killing to Gram-negative killing [11], and importantly, induced susceptibility and enhanced potencies against MDR bacteria [12]. Despite these astounding results, no siderophore–antibiotic conjugate as yet has been approved for clinical use.

**Figure 2:**
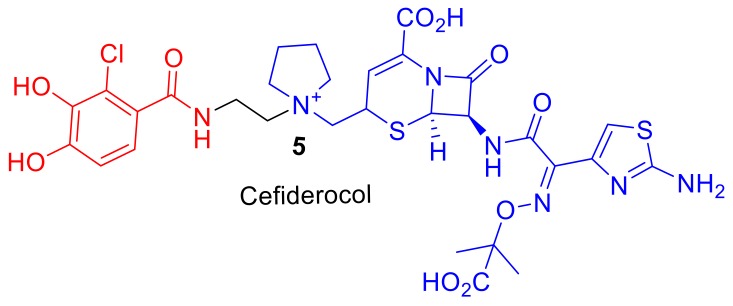
Cefiderocol, **5**, is in phase III clinical trials to treat Gram-negative pathogens [12]. Iron chelating catechol component represented in red and the cephalosporin antibiotic component in blue.

Cefiderocol (S-649266), **5**, (Figure 2), is a promising cephalosporin conjugate currently in phase III clinical trials [13]. Cefiderocol, **5**, can exhibit enhanced antibacterial potency in comparison to analogous third and fourth-generation cephalosporins such as ceftazidime and cefepime [12]. The major difference in chemical structure, to which the enhanced potency of cefiderocol, **5**, is attributed, is the presence of an iron-chelating catechol group. Cefiderocol, **5**, utilizes ferric iron transport uptake in Gram-negative pathogens [14]; it induces susceptibility and enhanced killing against antibiotic resistant strains of Gram-negative pathogens, including *Pseudomonas aeruginosa*, *Burkholderia cepacia*, *Acinetobacter baumannii*, and *Enterobacteriaceae* [12]. Therefore, molecules such as Cefiderocol, **5**, offer hope for the development of new siderophore–antibiotic conjugates as drugs to treat bacterial infections, including MDR bacteria. This review will explore reported siderophore–antibiotic conjugates and their design based on their subcomponents. However, an introduction into nature′s siderophore–antibiotics, sideromycins, is first required.

## 2. Sideromycins, Nature′s Siderophore–Antibiotic Conjugates

**Figure 3:**
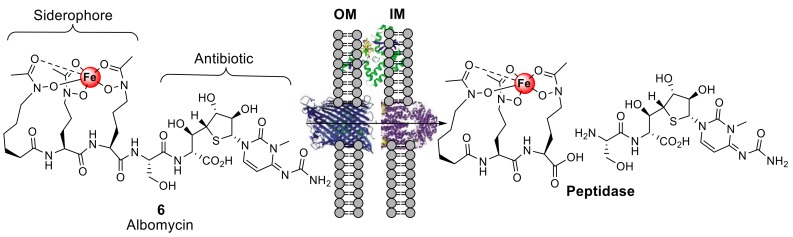
Naturally produced siderophore–antibiotic conjugate albomycin (**6**), and schematic diagram representing the mechanism for Gram-negative siderophore internalization. Albomycin is actively transported across the cell wall to the cytoplasm of Gram-negative bacteria such as *E. coli* by TonB-dependent transporter proteins [18]. Once in the cytoplasm, albomycin is cleaved by peptidases to release the antibiotic [15]. IM = Inner membrane, OM = Outer membrane; cartoon proteins represent TonB-dependent transport proteins.

Nature, which has provided most of the antibiotics on the market today, or derivatives thereof, also produces natural siderophore–antibiotic conjugates. Sideromycins are natural products consisting of an antibiotic conjugated to a siderophore; however, in comparison to natural product antibiotics, fewer sideromycins have been discovered, and the sideromycin albomycin, **6**, has been the most studied of the sideromycins to date (Figure 3) [15]. Gause played a leading role in the identification and antimicrobial studies of albomycin **6** [16], and remarkably, albomycin, **6**, was being used in the Soviet Union in the clinic as early as the 1940s and 1950s [16]. However, it was not until the 1980s that Benz et al., using synthesis, clarified the chemical structures of albomycins [17]. The two main components of albomycin, **6**, are a tri-hyrdroxmate iron-chelating component, which is directly analogous to the siderophore ferrichrome, and an antibacterial thioribosyl pyrimidine moiety (Figure 3). Albomycin, **6**, is internalized into Gram-negative species such as *Escherichia coli* by TonB-dependent transporters FhuA (outer membrane recognition), FhuD (periplasm), FhuB (cytoplasmic membrane), and FhuC, which is an ATPase that provides energy for transport across the inner membrane [18]. Once transported into the cytoplasm, albomycin is cleaved by a peptidase whereby the antibacterial moiety is released in the cytoplasm. The peptidase cleavage is essential to the antibacterial activity of the thioribosyl pyrimidine component of albomycin [15]. This active transport makes albomycin, **6**, a potent antibiotic with an MIC value 100-fold lower in comparison to the β-lactam antibiotic ampicillin in *E. coli* K-12 [19]. Recently, the natural product albomycins have succumbed to efforts in total synthesis, which may potentially lead to the development of active structural analogues [20]. Hence, albomycin and the sideromycins, nature′s natural siderophore–antibiotic conjugates, have provided inspiration for chemists in the design of synthetic siderophore–antibiotic conjugates.

## 3. Synthetic Siderophore-Antibiotic Conjugates

**Figure 4:**
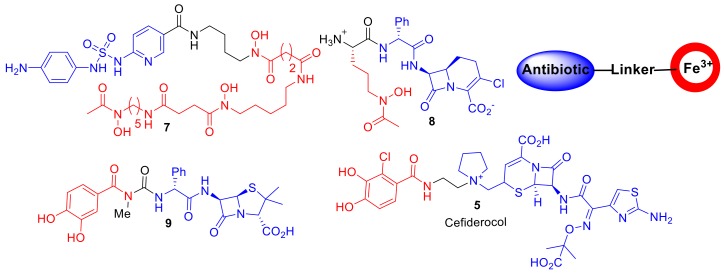
Compounds **7**–**9** represent a few of the earlier synthetic and semisynthetic siderophore–antibiotic conjugates. Cefiderocol, **5**, is currently in phase III clinical trials. The design of such synthetic conjugates has remained consistent with an antibiotic covalently attached to a Fe^3+^-chelating component via a linker (iron-chelating component in red and antibiotic in blue.).

Aside from derivatives of sideromycins, examples of ‘non-natural′ synthetic or semisynthetic siderophore–antibiotic conjugates were reported as early as the 1970s (Figure 4) [21]. Taking inspiration from sideromycins, Zahner et al. reported a semisynthesis of sulfonamide antibiotics covalently linked to ferrioxamine B analogues; however, compound **7** displayed only a limited spectrum of activity against Gram-positive *Staphylococcus aureus* [21]. Dolence et al. reported the total synthesis of carbacephalosporin β-lactam antibiotics conjugated to tripeptides such as compound **8** attempting to mimic the iron chelating functionality of albomycin [22]. A hypersensitive *E. coli* strain to β-lactam antibiotics exhibited delayed growth in the presence of **8**; the authors speculated that **8** was selecting for an iron transport-deficient mutant preventing the active uptake of **8**, and hence, only delayed bacterial growth was observed. However, the study provided important proof of the principle that antibiotics not naturally present in sideromycins could be transported into bacteria. Ohi et al. reported the synthesis of catechol analogues covalently linked to ureidopenicillins [23]. Such conjugates, including compound **9**, displayed a 30–60-fold decrease in MIC values compared to piperacillin against the opportunistic Gram-negative pathogen *P. aeruginosa* [23]. This work and related studies stimulated research into the catechol–β-lactam antibiotic conjugates in the 1980s and 1990s in academia and the pharmaceutical industry, which ultimately has led to the discovery of cefiderocol **5**. A comprehensive overview of the multiple analogues of β-lactam–siderophore conjugates can be found in the recent review by Lin et al. [24]. Over the years, the design of siderophore–antibiotic conjugates has not drastically changed since earlier examples; conjugates consist of an antibiotic covalently attached to an iron-chelating component via a linker moiety.

## 4. The Siderophore Component of the Conjugate

Many bacterial species are capable of recruiting cell surface proteins to transport non-endogenous siderophores for iron acquisition. Such siderophores, which are not endogenous to a producing microorganism but are recognized for uptake, are termed xenosiderophores [6]. This phenomenon is perhaps unsurprising, as despite the structurally diverse nature of siderophores, the iron-chelating functionalities generally remain consistent. This natural tolerance to siderophore structural diversity by bacteria for recognition and uptake is also true for synthetic siderophore–antibiotic conjugates. Studies to date indicate that the siderophore components in conjugates do not have to identically replicate natural siderophores [24]. In fact, enhanced uptake and MIC potencies have been reported in conjugates consisting of simplified mono-catechol components such as cefiderocol **5** [14] and artificial siderophores with multiple mixed Fe(III)-chelating functional groups [25]. However, despite this structural tolerance, many believe that replicating natural siderophores and their denticity is the most effective strategy for increasing the probability of developing a successful and truly effective synthetic conjugate [24,26]. Catecholates and hydroxamates make up the majority of the siderophore–antibiotic conjugates reported to date. Generally, catecholate moieties can provide a good starting point for siderophore–antibiotic conjugates, as the catecholate moiety is recognized by a number of bacterial species for uptake, particularly in Gram-negative bacteria [27], but also as a xenosiderophore by a number of Gram-positive bacteria [28], and even by *mycobacterium* spp. [29]. However, if this general approach is to be taken, and a specific bacterial species is the desired target of the conjugate, consideration must be made to the native siderophores produced by the targeted bacterial species. Such native siderophores may outcompete the siderophore–antibiotic conjugate for iron and render conjugate active transport and lowered MIC values redundant [30].

**Figure 5:**
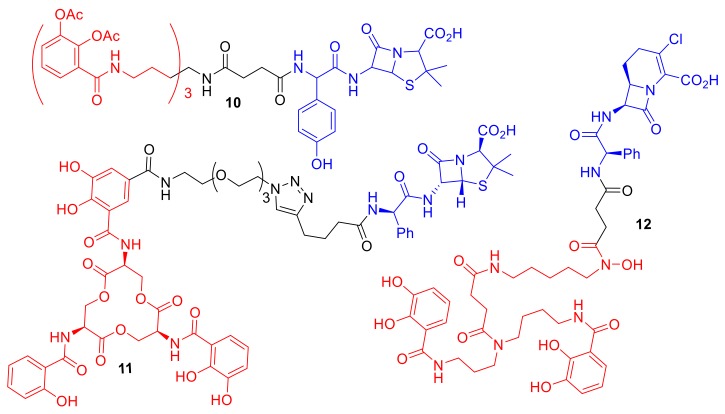
Synthetic catechol conjugates **10**–**12** that exhibit enhanced selectivity for specific bacterial species. **10** Minimum inhibitory concentrations (MICs): *P. aeruginosa* KW799 0.05 µM, *E. coli* ATCC 25,922 1.56 µM, *Klebsiella pneumoniae* ATCC 8303 × 68 > 100 µM [31]; **11** MICs: *E. coli* CFT073 10 nm, *K. pneumoniae ATCC* 13,883 > 100 µM, *P. aeruginosa* PA01 10 µM, *S. aureus* ATCC 25,923 10 µM [10]; **12** MICs: *A. baumannii* ATCC 17,961 7.80 nm, *S. aureus* SG511 32 µM, *E. coli* ATCC 25,922 32 µM [25].

One exciting observation with regard to synthetic siderophore–antibiotic conjugates is that the siderophore component can potentially be utilized to gain lowered MIC values and selectivity for a specific bacterial species (Figure 5). Ji et al. reported the synthesis of a tris-catechol enterobactin mimic conjugated to the β-lactam antibiotic amoxicillin, conjugate **10** [31]. Conjugate **10** demonstrated enhanced potencies against Gram-negative bacteria and in particular against *P. aeruginosa* over other Gram-negative bacterial species. Acetyl groups were installed on the catechol hydroxyl groups in the siderophore component of **10** to act as a pro-drug preventing catechol metabolism and modification in vivo. Interestingly, although conjugate **10** exhibited selectivity for *P. aeruginosa* strains, one *P. aeruginosa* clinical isolate strain, known as PA6, was not susceptible to **10**. This finding suggests that the PA6 strain may utilize a modified iron uptake system to other *P. aeruginosa* strains, or alternatively may overexpress beta-lactamases, rendering the antibiotic in the conjugate ineffective. A ciprofloxacin conjugate utilizing the same tris-catechol enterobactin mimic as **10**, but lacking the acetyl groups and containing a differing linker, was reported by Fardeau et al. [32]. The conjugate by Fardeau et al. displayed only moderate antibacterial activity against *P. aeruginosa*, and the MIC was not significantly lowered compared to ciprofloxacin alone.

Zheng et al. functionalized the natural siderophore enterobactin to ampicillin, making conjugate **11** (Figure 5) [10]. This conjugate demonstrated a 1000-fold reduction in MIC compared to ampicillin in *E. coli* strains. Conjugate **11** was capable of selectively killing *E. coli* even when co-cultured with Gram-positive *S. aureus*. The authors highlighted the use of the natural siderophore enterobactin, rather than an artificial analogue, leading to the observed species selectivity and enhanced potency of **11**. The species selectivity of conjugate **11** compared to conjugate **10** is intriguing, considering that **10** is a mimic of enterobactin; this difference perhaps highlights the differences in structural recognition by the TonB-dependent transport proteins between *E. coli* and *P. aeruginosa*, with *E. coli* exhibiting a preference for the more rigid tri-peptide scaffold of enterobactin conjugate **11** over the mimic **10**. In another study by Nolan et al., an analogue of conjugate **11** was synthesized containing an additional sugar group on the catechol to mimic the natural siderophore salmochelin [33]. The sugar-functionalized conjugate exhibited reduced MIC values for pathogenic *E. coli* strains that utilize the salmochelin receptor IroN, demonstrating not only bacterial species selectivity for *E. coli,* but also enhanced selectivity for pathogenic *E. coli* strains over non-pathogenic *E. coli*.

Wencewicz and Miller utilized an artificial mixed bis(catechol) and hydroxmate siderophore mimic in conjugate **12** (Figure 5) [25]. This artificial siderophore had previously been reported to have unexpected selectivity for the Gram-negative pathogen *Acinetobacter baumannii* [34]. The conjugate exhibited an MIC of 7.80 nM against *A. baumannii* that was dependent upon iron concentration, while the parent antibiotic from which conjugate **12** was derived had an MIC greater than 128 µM in *A*. *baumannii*. The conjugate demonstrated moderate antibacterial activity against *S. aureus* and *E. coli* (MICs: 32 µM and 8 µM respectively).

Conjugates **10**, **11**, and **12** based on catechol siderophores, or mixed hydroxmate–catechol siderophores, demonstrate that catechols can be important components for inducing Gram-negative species selectivity. Additionally, when considering conjugates **10**, **11**, and **12**, it is important to note that **10** was designed to mimic enterobactin, **11** utilizes enterobactin, and **12** was designed to mimic the natural siderophore fimsbactin A [25,34]. Hence, the design of all three of these highly effective conjugates were based on natural siderophores.

**Figure 6:**
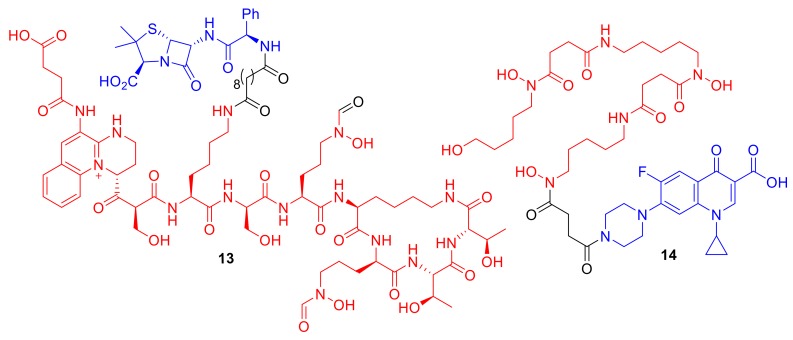
Synthetic conjugates **13** and **14** exhibit enhanced selectivity for specific bacterial species. **13** MICs: *P. aeruginosa* PA01 0.39 µM, *P. aeruginosa* ATCC 27,853 > 100 µM [36]; **14** MICs: *S. aureus* SG 511 1 µM, *A. baumannii* ATCC 17,961 > 128 µM, *P. aeruginosa* ATCC 27,853 > 128 µM, *E. coli* ATCC 25,922 > 128 µM [38].

Kinzel et al. synthesized conjugates of *Pseudomonas* pyoverdine siderophores to ampicillin creating conjugates such as **13** (Figure 6) [35]. The pyoverdine conjugates, including **13**, demonstrated potent antibacterial activity in the strains of *P. aeruginosa* from which the pyoverdines were naturally derived. Remarkably, **13** exhibited an MIC of 0.39 µM against its native pyoverdine-producing PA01 strain, which is significantly lower than its MIC values against the non-pyoverdine-producing *Pseudomonas* strain ATCC 27,853 (>100 µM); these conjugates are clearly utilizing the siderophore counterpart′s native iron transport mechanisms for active uptake. Conjugate **13** demonstrates, yet again, how utilizing natural siderophores can result in effective conjugates. Other synthetic pyoverdine-based β-lactam and DNA gyrase inhibitor conjugates have met with mixed results; these compounds generally exhibit reduced antibacterial activity compared to the parent antibiotic from which they were derived [36,37].

Wencewicz et al. synthesized conjugate **14**, which contains a siderophore directly analogous to the natural siderophore desferrioxamine B attached to the quinolone antibiotic ciprofloxacin targeting DNA gyrase [38]. The conjugate did not display enhanced potency compared to ciprofloxacin alone; however, the conjugate did exhibit enhanced selectivity toward *S. aureus*. The authors demonstrated that only the trihydroxamate functionality, mimicking natural desferrioxamine B, was responsible for this observed *S. aureus* species selectivity. Desferrioxamine B is already used in patients to treat iron overload; therefore, it could be envisaged that conjugate **14** could have applications in vivo in patients with *S. aureus* infections. Conjugate **14** is also an example of how hydroxamate siderophores can be utilized in conjugates to gain Gram-positive species selectivity. Milliner et al. sought to gain selective antibacterial activity against *S. aureus* by conjugating a number of analogues of staphyloferrin A, which is a natural siderophore of *S. aureus*, to ciprofloxacin [39]. However, these conjugates exhibited reduced antibacterial potencies compared to ciprofloxacin alone, and the authors demonstrated that the functionalization of ciprofloxacin compromised DNA gyrase inhibition and proposed this to be responsible for the reduced antibacterial activity of the conjugate.

## 5. The Linker Component of the Conjugate

Many functional groups have been incorporated into synthetic conjugates to form the basis of the linker, including but not limited to: amino acids [22], succinic acid [39], alkyl chains [14], polyethylene glycol (PEG) chains [10], triazoles [40], acetal groups [41], disulfide bonds [42], thiol-maleimides [43], citric acid [44], esters [45], and even β-lactam antibiotics [46]. However, linkers are generally categorized based on whether they have been specifically designed to be cleavable or non-cleavable. Cleavage can occur by different mechanisms, including by hydrolysis [41], enzyme-catalyzed cleavage [47], and reduction [42]. To date, it has been found that synthetic conjugates consisting of β-lactam antibiotics generally do not require a cleavable linker for the active transport and enhanced MIC potencies. One hypothesis for the effectiveness of non-cleavable β-lactam conjugates is that the target of β-lactam antibiotics, penicillin-binding proteins, are localized in the periplasm of the bacterial cell wall; therefore, active transport into the bacterial cytoplasm is not necessary for antibacterial activity [48]. This hypothesis has been further evidenced by the observation that synthetic conjugates consisting of antibiotics with targets in the cytoplasm require cleavage to at least retain MIC values comparable to the parent antibiotic from which they were derived [49,50]. Hence, synthetic conjugates targeting cytoplasmic proteins are required to reach the cytoplasm and also, in cases to date, are required to undergo cleavage in the cytoplasm to engage the intracellular antibiotic target.

There is no doubt that designing effective conjugates with cleavable linkers for intracellular targets, mimicking nature′s sideromycins, has proved much more challenging for researchers. The cleavable linker must be stable toward the extracellular environment, but cleaved upon entry into the cytoplasm. A linker that is too stable will attenuate the activity of the antibiotic, while linkers that are more susceptible to cleavage will result in premature antibiotic release; in both scenarios, the benefits of a conjugate are lost. Hence, a ‘fine balance′ of chemical reactivity is required for a cleavable linker, and a number of studies utilizing cleavable linkers have been unsuccessful.

**Figure 7:**
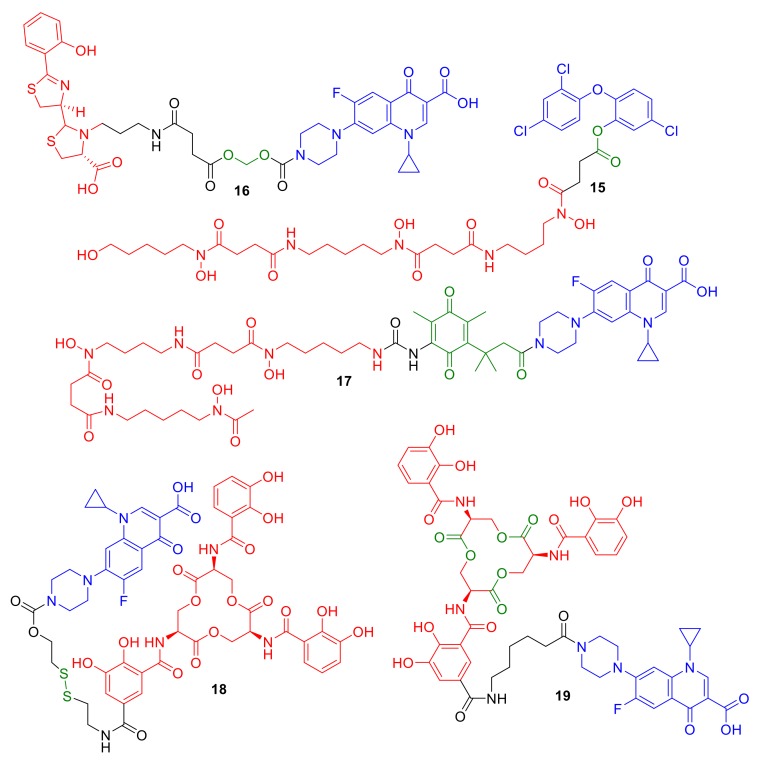
Conjugates **15**–**19** represent synthetic conjugates designed to incorporate cleavage mechanisms for cytoplasm delivery. Intended cleavage-initiating functional groups are highlighted in green.

Using esters to exploit antibiotic release from conjugates has been previously reported [26,45,47]. Taking inspiration from nature and the sideromycin desferrisalmycin B, Wencewicz et al. synthesized a desferrioxamine–triclosan conjugate **15** (Figure 7) [49]; triclosan exhibits its antibacterial activity by inhibiting fatty acid biosynthesis in bacteria. The phenolic ester linkage was reported to be important for the antibacterial activity of conjugate **15**, which was comparable to triclosan alone and was enhanced in comparison to similar conjugates with an amide linkage. However, unexpected antibacterial activity against *E. coli*, which was speculated to be non-siderophore mediated, led the authors to speculate that the conjugate was undergoing premature cleavage in the extracellular environment. A number of studies have investigated the use of acetal functional groups for hydrolysis-mediated linker cleavage [40,45,51]. The ciprofloxacin–pyochelin conjugate **16** was synthesized by Noël et al. with a cleavable acetal linker [51]. The pyochelin siderophore was utilized in the conjugate in an attempt to gain enhanced antibacterial activity against *P. aeruginosa* with ciprofloxacin targeting the cytoplasm-localized DNA gyrase. Although cleavable conjugate **16** displayed antibacterial activity that was not exhibited by non-hydrolysable linker analogues, the conjugate was less active than the parent antibiotic ciprofloxacin alone. The authors highlighted solubility issues with conjugate **16**, while premature hydrolysis in the extracellular environment was also highlighted to be a significant issue with these conjugates. This premature hydrolysis is a reported problem for many other acetal-containing linkers in conjugates.

Ji and Miller investigated linking the antibiotic to siderophores via a ‘trimethyl lock′ in conjugate **17** [52]. The antibiotic release of **17** was designed to be triggered by the reduction of the benzoquinone functionality, followed by lactonization, and subsequent antibiotic release, intended to occur during Fe(III) to Fe(II) reduction in the bacterial cell [52]. The desferrioxamine B–ciprofloxacin conjugate **17** in this study demonstrated moderately reduced MICs in comparison to a directly analogous non-cleavable counterpart. However, the conjugate was not as potent as ciprofloxacin itself, suggesting that more optimization of the cleavage mechanism of **17** is required. Similar results were observed by Miller et al. this time using an esterase-cleavable trimethyl lock [47]; these conjugates exhibited antibacterial activity, but were not as potent as the parent antibiotic ciprofloxacin from which they were derived. Other reducible linker strategies such as that employed in conjugate **18** have been reported by Neumann and Nolan [42]. Again, ciprofloxacin is the antibiotic of choice conjugated to enterobactin, while a disulfide bond is employed as the intended cleavage site. Mixed results were reported for **18**, which exhibited antibacterial activity against only a limited number of *E. coli* strains. The authors concluded that disulfide-containing linkers may not be a broad strategy for conjugate deign.

Despite all these challenges, a significant advancement in cleavage methodologies has recently been reported by Neumann et al. [26]. The authors designed and synthesized enterobactin–ciproloxacin conjugate **19**, which was designed to be susceptible to intracellular enzymatic cleavage. The conjugate **19** could perhaps be considered as a siderophore-mediated cleavable conjugate rather than a linker-mediated cleavable conjugate. The catecholate siderophores enterobactin and salmochelin are internalized naturally in Gram-negative bacteria such as *E. coli* and undergo cleavage to a number of siderophore subfragments; Neumann et al. used these observations as inspiration for the conjugate design of **19**. Conjugate **19** was designed to have an amide bond on enterobactin in the same position as the glycodside linkage in the analogous siderophore salmochelin. Salmochelin undergoes intracellular enzyme-mediated hydrolysis by the esterase IroD. Remarkable results were observed with conjugate **19**, which was not only as active as its parent antibiotic (a real achievement for a cytoplasmic-targeting conjugate), but demonstrated selective antibacterial activity against pathogenic *E. coli* over non-pathogenic *E. coli.* This selectivity was rationalized by esterase IroD being predominantly present in pathogenic *E. coli* in comparison to non-pathogenic *E. coli.* There are other significant observations in cleavable conjugate design in this study that should not be overlooked. (1) A compound directly analogous to **19** with a longer PEG linker instead of the alky chain of **19** was not active against any of the tested *E. coli* strains. (2) It could be assumed a modified analogue of ciprofloxacin, such as a linker or siderophore subfragment analogue of ciprofloxacin, was released that was capable of retaining DNA gyrase inhibition and antibacterial activity. Hence, the chemical make-up of the linker is just as important as the cleavage mechanism/design, and although cleavage is necessary, an analogue of the cleaved antibiotic may be tolerated to retain antibiotic target engagement. Such a discovery offers further hope and research for cleavable linkers, and perhaps further highlights the use of natural siderophores in conjugates to mimic nature more closely for success.

## 6. The Antibiotic/Antimicrobial Component of the Conjugate

Synthetic conjugates consisting of β-lactam antibiotics are the most reported in the literature to date followed secondly by DNA gyrase inhibitors such as ciprofloxacin. Exciting results have been observed with β-lactam conjugates enhancing MIC potencies and inducing multi-drug resistant (MDR) bacterial susceptibility. Ciprofloxacin is a broad spectrum antibiotic that is widely used to treat a number of bacterial infections; with a low molecular weight (331 Da) and a readily functionalized piperazine heterocycle, this antibiotic provides a robust chemical scaffold for investigating conjugate delivery to the cytoplasm. Many other antibiotics have been conjugated to siderophores, with conjugates **6** and **15** in this review being good examples. However, antibiotics other than β-lactams and DNA gyrase inhibitors have not been as well represented in recent decades. Considering the extensive literature on β-lactam and ciprofloxacin conjugates [24,53,54,55], which also make up most of the conjugate examples so far in this review, this section focuses on selected examples of non-β-lactam and non-ciprofloxacin conjugates.

**Figure 8:**
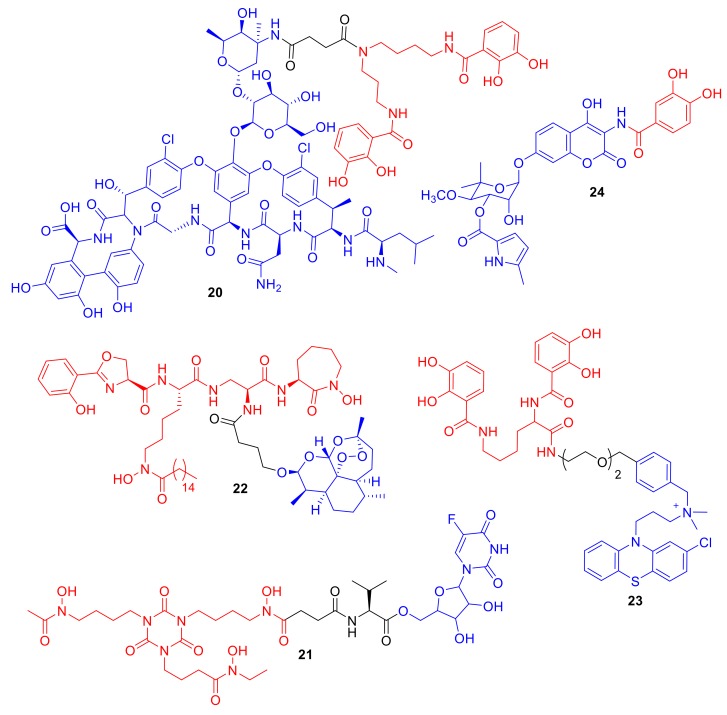
Conjugates **20**–**24** are representative examples of non-β-lactam and non-ciprofloxacin conjugates.

Miller et al. conjugated the Gram-positive antibiotic vancomycin to catechol ligands and mixed catechol–hydroxmate ligands [56]; however, MIC values were generally higher against Gram-positive bacteria compared to vancomycin alone, and were comparable to those of vancomycin against Gram-negative bacteria. Vancomycin conjugate **20** (Figure 8) did exhibit reduced MIC values in a hypersensitive *P. aeruginosa* strain over vancomycin alone. Gosh et al. conjugated the active metabolite of the antifungal agent 5-fluorocytosine (5-FC) to trihydroxamate siderophore mimics as well as to norneoenactin and an erythromycin analogue [57]. The erythromycin conjugate did not exhibit any significant antibacterial activity, and the authors hypothesized that erythromycin was too large for siderophore-mediated delivery. The 5-FC conjugate **21**, which was functionalized for ester-mediated cleavage, did display enhanced antibacterial activity compared to the 5-FC active metabolite alone against Gram-positive *Staphylococcus* and *Enterococcus* spp. In a related study, Lu and Miller carried out an interesting modification to the ‘typical′ siderophore–antibiotic conjugate design; the authors functionalized the conjugate with multiple 5-FC metabolite antibacterial groups [58] rather than the mono-antibiotic functionalization motif typically used in conjugates. The conjugates again exhibited enhanced antibacterial activity compared to 5-FC alone, and the multi-5-FC conjugates displaying enhanced potency over mono 5-FC conjugates.

In another elegant study by Miller et al., an acetal analogue of the antimalarial agent artemisinin was conjugated to an analogue of the siderophore mycobactin T, which is utilized by *Mycobacterium tuberculosis* [59]. The difficulty of synthesizing conjugate **22** should not be underestimated, as the synthetic route involves the synthesis of both the mycobactin T scaffold and the endoperoxide 1,2,4-trioxane ring of artemisinin. The biological results were also impressive; the attachment of mycobactin T appeared to convert artemisinin from an exclusively antimalarial agent into a dual potent anti-tuberculosis (TB) agent, with MICs ranging from 0.125–1.25 µg/L against eight differing MDR *M. tuberculosis* clinical isolates. The authors hypothesized that microbial Fe(III) reduction to Fe(II) leads to the conversion of artemisinin into the reduced redox state; the reduced artemisinin induces the production of oxygen free radicals that invoke antimicrobial activity. Therefore, in this case, the siderophore not only enhances delivery and uptake, but also enhances the mode of action of the antimicrobial agent. Within our group, we recently reported the synthesis of a catechol azotochelin–phenothiazine conjugate **23** [60]. The phenothiazine used targets membrane-localized type II NADH:quinone oxidoreductase (NDH-2), which is an ATP synthase that is important for the survival of TB and a number of Gram-positive bacterial species [61]. NDH-2 is also the secondary target of the antibiotic colistin against Gram-negative bacteria [62]. As NDH-2 is localized in the cell wall, similar to β-lactam conjugates targeting penicillin binding proteins (PBPs), we used a non-cleavable linker in our design. Alt et al. prepared the unique conjugate **24** utilizing synthetic biology rather than chemical synthesis [63]. Conjugate **24** contains an analogue of the DNA gyrase inhibitor clorobiocin. The conjugate utilizes TonB-dependent active transport and demonstrates enhanced antibacterial activity compared to clorobiocin in TolC efflux pump mutants. However, conjugate **24** was not as active as clorobiocin against *E. coli* K12; the authors suggest that, although the active uptake of **24** is increased compared to clorobiocin alone, so too is the TolC-mediated active efflux of conjugate **24**.

**Figure 9:**
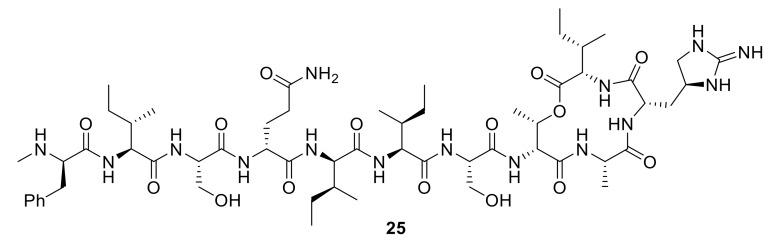
Teixobactin, **25**, a new antibiotic targeting lipid II and lipid III, demonstrating activity against Gram-positive bacteria [64].

**Figure 10:**
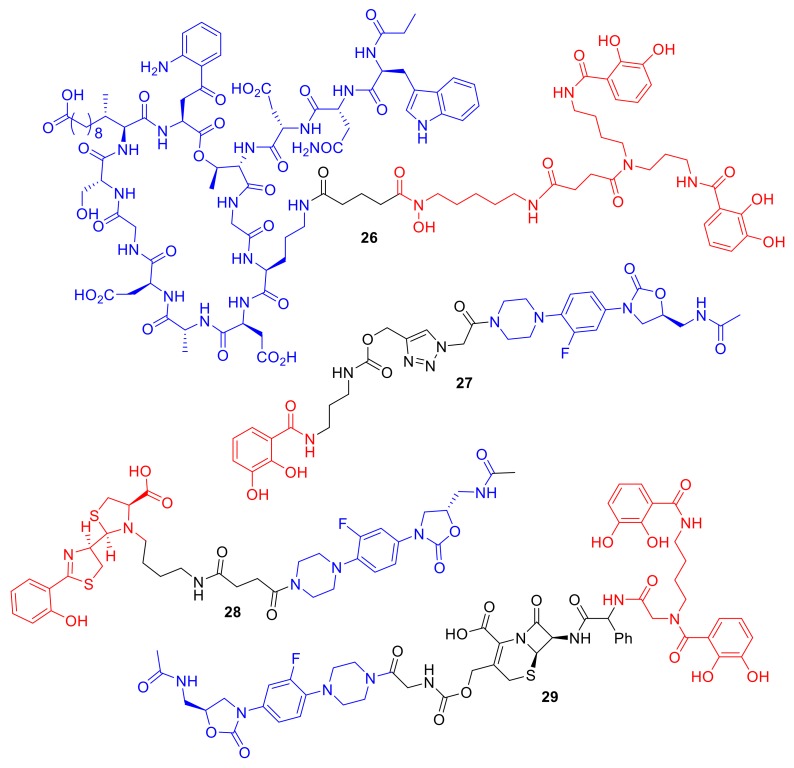
Conjugates **26**–**29** are representative examples of conjugates transforming Gram-positive antibiotics to induce Gram-negative killing.

One area of siderophore–antibiotic research that has readily driven new conjugates is the desire to generate new molecules to treat Gram-negative pathogens. The additional lipopolysaccharides in their outer membranes, which are not present in Gram-positive bacteria, present a formidable barrier to the penetration and diffusion of antibiotics into Gram-negative bacterial cells [5]. Hence, the development of new antibiotics for Gram-negative bacteria is more challenging and of grave concern for new antibiotic discovery; for example, the new antibiotic teixobactin, **25**, (Figure 9), is not active against Gram-negative bacteria [64]. Hence, siderophore-mediated antibiotic delivery via conjugates offers an exciting approach to turn known Gram-positive antibiotics into Gram-negative antibiotics. Using a mixed catechol and hydroxmate siderophore analogue, mimicking the natural siderophore fimsbactin of Gram-negative *A. baumannii*, Gosh et al. turned the Gram-positive antibiotic daptomycin into a potent killer in vitro and in vivo of *A. baumannii* [65]. The activity of conjugate **26** (Figure 10) was dependent on the iron-chelating functionalities of the siderophore, and MIC values were exhibited of 0.4 µM and 12.5 µM against *A. baumannii* ATCC 17,961 and *S. aureus* ATCC 11,632, respectively. In comparison, daptomycin alone has MIC values of 0.4 µM against *S. aureus* ATCC 11,632 and greater than 100 µM against *A. baumannii* ATCC 17961. Remarkably, the study highlighted that large antibiotics (daptomycin; 1630 Da) can indeed be functionalized for siderophore-mediated delivery. In another approach, Paulen et al. synthesized an analogue of linezolid, one of the newer antibiotics in the market, to catecholates with the aim of lowering the MICs of this Gram-positive antibiotic against *P. aeruginosa* [40]. Conjugate **27** exhibited an MIC of 128 µM against *P. aeruginosa*, which is significantly lower than that of linzeloid alone (>1024 µM); this is an intriguing result, as the linker was not designed to be cleavable, and linzeloid′s antibiotic target is in the cytoplasm. The activity of conjugate **26** was also enhanced in iron-deficient growth medium, suggesting the iron transportation of the conjugate.

In a different study, Paulen et al. conjugated linzeloid to the *P. aeruginosa* siderophore pyochelin, forming conjugate **28** as well as analogues (Figure 10). Unfortunately, all the conjugates in this study demonstrated little or no antibiotic activity [66]. The authors noted that the conjugates were plagued with solubility issues; similar issues were reported with pyochelin conjugates such as **16** prepared by Noël et al. [51]. A rather unique linzeloid conjugate **29** synthesized by Liu et al. consisted of linezolid conjugated to a catechol siderophore; however, the linker was a cephalosporin β-lactam antibiotic [46]. The authors hypothesized that, on bacterial cell entry, the conjugate would be cleaved by penicillin-binding proteins or β-lactamases, thus releasing linzeloid into the bacterial cell. Conjugate cleavage would presumably be increased in antibiotic-resistant β-lactamase-producing strains. The MIC of conjugate **29** was 125-fold more potent than cephalosporin alone and the conjugate lacking the β-lactam linker against *A. baumannii*, which indicates that the penicillin-binding protein or β-lactamase cleavage is important for the observed enhancement of antibacterial activity. The cephalosporin-linked conjugate **29** was active against all four clinical isolates of *A. baumannii* that it was tested against.

## 7. Conclusions and Future Outlook

The advantages that can potentially be offered by siderophore–antibiotic conjugates are clear; enhanced antibacterial potency, induced bacterial species selectivity, and even enhanced selectivity for pathogenic strains over non-pathogenic strains, transforming Gram-positive antibiotics into potent Gram-negative antibiotics, and rendering MDR pathogenic bacteria susceptible to killing. These advantages are further exemplified by cefiderocol, **5**, in phase III clinical trials with potent activity against MDR Gram-negative bacteria [13]. If cefiderocol, **5**, progresses to the market, further drug discovery research into siderophore–antibiotic conjugates would most likely be stimulated. Indeed, β-lactam–siderophore conjugates are already in the realm of medicinal chemistry and drug discovery. However, non-β-lactam siderophore–antibiotic conjugates seem to remain, as yet, an academic pursuit. Perhaps such large molecular weight conjugates may put medicinal chemists off the drug discovery process. Despite this, importantly, caution needs to be taken in overlooking such larger conjugates, since if there is one field in medicinal chemistry that perhaps ‘tolerates′ bigger molecules and violations of Lipinski′s rules, it is natural product antibiotics [67]. Further to this, the siderophore desferrioxamine B (mwt 560 Da) is already used in the clinic to treat patients for iron overload.

It would be interesting to see more conjugates utilizing antibiotics and antimicrobial agents other than the β-lactam antibiotics and DNA gyrase inhibitors that currently dominate the field. Linzeloid conjugates are becoming more common. Further to this, it would be interesting to see more conjugates utilizing more structurally diverse natural siderophores, considering that over 500 siderophores have been reported [6]. Given the recent success in cleavable conjugates reported by Nolan et al. [26], there is also a need to revisit the synthesis of natural siderophores and to conduct further biological studies on their active transport and iron delivery mechanisms. The potential chemical space of siderophore–antibiotic conjugates is vast and can be considered, as yet, an untapped resource for the generation of desperately needed new antibiotics. However, it is also important to note such conjugates should not be considered a ‘magic bullet′ to antibiotic resistance, but rather important molecules and potential medicines to add to our current antibiotic arsenal.
